# Centrifugal Pump Fault Diagnosis Based on a Novel SobelEdge Scalogram and CNN

**DOI:** 10.3390/s23115255

**Published:** 2023-06-01

**Authors:** Wasim Zaman, Zahoor Ahmad, Muhammad Farooq Siddique, Niamat Ullah, Jong-Myon Kim

**Affiliations:** 1 Department of Electrical, Electronic and Computer Engineering, University of Ulsan, Ulsan 44610, Republic of Korea; wasim94@mail.ulsan.ac.kr (W.Z.); zahooruou@mail.ulsan.ac.kr (Z.A.); mfarooq229@mail.ulsan.ac.kr (M.F.S.); niamat016@mail.ulsan.ac.kr (N.U.); 2 PD Technology Cooperation, Ulsan 44610, Republic of Korea

**Keywords:** centrifugal pump, stockwell transform, fault diagnosis, rotating machinery, convolutional neural network, vibrational signals

## Abstract

This paper presents a novel framework for classifying ongoing conditions in centrifugal pumps based on signal processing and deep learning techniques. First, vibration signals are acquired from the centrifugal pump. The acquired vibration signals are heavily affected by macrostructural vibration noise. To overcome the influence of noise, pre-processing techniques are employed on the vibration signal, and a fault-specific frequency band is chosen. The Stockwell transform (S-transform) is then applied to this band, yielding S-transform scalograms that depict energy fluctuations across different frequencies and time scales, represented by color intensity variations. Nevertheless, the accuracy of these scalograms can be compromised by the presence of interference noise. To address this concern, an additional step involving the Sobel filter is applied to the S-transform scalograms, resulting in the generation of novel SobelEdge scalograms. These SobelEdge scalograms aim to enhance the clarity and discriminative features of fault-related information while minimizing the impact of interference noise. The novel scalograms heighten energy variation in the S-transform scalograms by detecting the edges where color intensities change. These new scalograms are then provided to a convolutional neural network (CNN) for the fault classification of centrifugal pumps. The centrifugal pump fault classification capability of the proposed method outperformed state-of-the-art reference methods.

## 1. Introduction

Centrifugal pumps (CPs) are gaining popularity in numerous technological applications, including engine manufacturing, air conditioning, chemical processing, and electricity generation [1]. Around 20% of the total energy produced worldwide is consumed by motors driving CPs [2,3]. Despite the long lifespan of CPs, their abrupt failure can cause unwanted interruptions or even catastrophic failures. These failures lead to economic losses, long downtime, and costly repairs. To avoid these failures, continuous monitoring of CPs is required. Monitoring can be provided either by several staff members or by signal processing and artificial intelligence (AI) techniques that are relatively low-cost and more reliable [3]. Intelligent Fault Diagnosis must be used to quickly detect soft faults. 34% of the CP soft faults are caused by faults with the mechanical seal (MS). Soft CP faults, such as fluid flushing, shaft wear, fretting, etc., are caused by defective MS. Additionally, a flawed impeller might result in both mechanical and hydraulic soft faults [4]. For this reason, in recent years AI-based condition monitoring is receiving increasing attention. Mechanical faults such as mechanical seal-related defects and impeller defects can cause catastrophic failures in the CP. To avoid catastrophic failure, minimize downtime, and ensure the safety and efficiency of the production system, it is essential to immediately identify and diagnose these defects in the CPs.

In the past decade, condition-based monitoring (CBM) gained popularity for the health monitoring of CPs. CBM is based on data gathered from the machine under various situations, making it an economical means of increasing a machine’s runtime [5].

Different types of faults are possible in CPs, namely hydraulic and mechanical faults. Although these faults are interdependent mechanical failures happen more often [6]. Thus, mechanical faults such as impeller faults, mechanical seal holes, and scratches must be detected early to maintain a CP’s health. The vibration signals received from a CP are significantly impacted by mechanical faults; these faults cause the vibration signals to be impulsive and non-stationary, which demands attention to analyze these vibration signals for fault diagnosis [7]. Vibration signals must be pre-processed to extract relevant fault-related features since fault-related features are frequently obscure and disguised by the signals’ considerable amounts of noise and fading fault impulses. Time, frequency, and time-frequency-domain (TFD) analyses are the three basic methods for signal processing.

The Fourier transform (FT), one of several signal processing techniques, is the most widely used in the applications of stationary signals. However, because of the loss of temporal data, both discrete and continuous FTs yield inaccurate information for non-stationary signals even though they still retain spectral component information [8]. To successfully analyze non-stationary data, new signal processing techniques such as the short-term Fourier transform (STFT), wavelet transform (WT), and Stockwell transform (S-transform) have been introduced [9]. The STFT utilizes fixed sample windows, which are often used for time-frequency analysis; however, higher time resolution might result in lower frequency resolution and vice versa [10]. In contrast, to address the STFT’s resolution issues, the WT employs larger windows at lower frequencies and smaller windows at higher frequencies and is highly successful at collecting information in the time and frequency domains [11]. However, the WT is noise-sensitive and lacks phase information for the analyzed signals [12]. The WT has gained increasing attention in recent years for its efficacy in processing nonstationary signals, as evidenced by several studies [13]. Extensive research over the past couple of decades has been devoted to exploring its utility in machine condition monitoring and health diagnostics [14]. WTs have been employed successfully in diverse applications, such as bearing condition monitoring [15,16,17], detection of machine tool failure [18], detection of knock and misfire in spark ignition engines [19], fault detection in washing machines [20], and monitoring of alternating-current drives [21]. An energy-based approach for selecting the optimal base wavelet and ideal decomposition scale using the energy content of the signal’s wavelet coefficients as a criterion was proposed by Ruqiang et al. [22], whereby the envelopes of the extracted features were subsequently subjected to Fourier transform to identify the presence and location of defects in rotating machinery. The methodology proposed by Delgado et al. [23] for bearing fault diagnosis involved analyzing significant characteristics from a feature set obtained by statistical-time features computed from vibration signals, applying a nonlinear manifold learning technique for dimensionality reduction, and performing classification using a hierarchical neural network. In 2017, Xia et al. used a convolutional neural network (CNN)-based approach for the diagnosis of rotating machinery, which involved collecting vibration data from multiple sensors, combining the data using a data fusion process into one 2D matrix, and training a CNN model on the extracted features for automatic feature representation [24]. A three-phase technique proposed by Ahmad et al. [25] in 2021 involved the transformation of CP vibration signature using the Walsh Transform in the first phase, extraction of raw statistical features in the time and Walsh Spectrum domain in the second phase, and the use of cosine linear discriminant analysis (CLDA) in the final step to choose comparable interclass properties and incorporate them into the final feature pool, which was then fed into the KNN algorithm for fault classification. A fault diagnosis technique for multistage centrifugal pumps (MCP) was proposed by Ahmad et al. [4], where informative ratio principal component analysis (Ir-PCA) was utilized for dimension reduction in the features extracted from the fault-specific frequency band of the vibration signal from the CP, which included statistical features in the time and frequency domains as well as features extracted from the wavelet domain and fed into a multi-domain feature pool (MDFP) for classification using the KNN algorithm. Sajjad et al. [26], proposed a fault classification technique for the CP that involves visualizing fault-related impulses through kurtogram spectra computation and training a convolution encoder with a supervised contrastive loss followed by training a linear classifier over the frozen encoder for fault classification.

The abovementioned work shows better performance for CP fault diagnosis; however, several shortcomings exist. First, extracting handcrafted features from the vibration signals requires domain expertise. Second, mother wavelet selection for WT entails experimentation and domain knowledge. To overcome these shortcomings, this paper proposes a new framework for CP fault diagnosis. The framework employs the S-transform for preprocessing the CP vibrational signals, which overcomes the limitations of both STFT and WT by preserving a close connection to the Fourier spectrum, giving frequency-dependent resolution. After the transformation, a Sobel filter is applied to the traditional scalograms, and novel SobelEdge scalograms are obtained. The novel scalograms enhance the energy variations in the S-transform scalograms by detecting the edges where color intensities change. These new scalograms are then provided to CNN for the classification of centrifugal pump health conditions.

The overall contribution of the proposed work can be summarized as follows:

1. A low-pass filter is applied to vibrational signals to extract fault-specific frequencies to improve signal quality and eliminate high-frequency noise.
2. The S-transform is employed to increase time-frequency resolution, enabling a more precise study of the frequency content of a signal across time. This transformation is favored over alternative time-frequency representations, such as the short-time Fourier transform or wavelet transform, due to its superior resolution and capacity to handle non-stationary signals.
3. The Sobel filter is used to preprocess the Stockwell scalograms; as a result, novel SobelEdge scalograms are obtained. The novel scalograms enhance the energy variations in the S-transform scalograms by detecting the edges where color intensities change that may be indicative of significant signal occurrences such as transients and abnormalities. To the best of the author’s knowledge, the SobelEdge Scalograms have not been reported in the literature previously.
4. CNN is used to extract features with substantially more pronounced classification, which may be used for fault classification. CNNs are trained on SobelEdge Scalograms to acquire features that are discriminative and invariant to signal fluctuations. By merging diverse signal processing and deep learning approaches to enhance the accuracy and dependability of the analysis, the proposed method provides a complete solution for defect detection and condition monitoring.

This paper is structured as follows: the proposed method is discussed in Section 2, the experimental setup and test rig setup for the centrifugal pump are described in Section 3, the technical background of the proposed method is discussed in Section 4, Section 5 comprises results and discussion, and Section 6 concludes with a summary and suggestions for future research.

## 2. Proposed Approach

In the area of vibration analysis, the S-transform is a potent tool for studying non-stationary signals. By offering a localized spectral estimate of the signal, it is a time-frequency analysis technique that enables a more precise representation of the frequency content of a signal across time.

The S-transform can be very helpful in the context of centrifugal pump vibration analysis for identifying and diagnosing faults with the pump’s rotating parts, such as the impeller and mechanical seal. S-transform works well in identifying fault types such as impeller faults, mechanical seal holes, and mechanical seal scratches as well as differentiating between healthy and unhealthy circumstances. However, these scalograms still contain noise.

The Sobel filter is used on the Stockwell scalograms in order to enhance the details of the scalograms’ edges. The Sobel filter is often used in image processing for the purpose of edge detection. When the Sobel filter was applied to Stockwell scalograms, the outcome was the development of Sobel-filtered Stockwell scalograms. These scalograms allowed for improved visibility of the edges that were present in the original scalograms.

**Figure 1:**
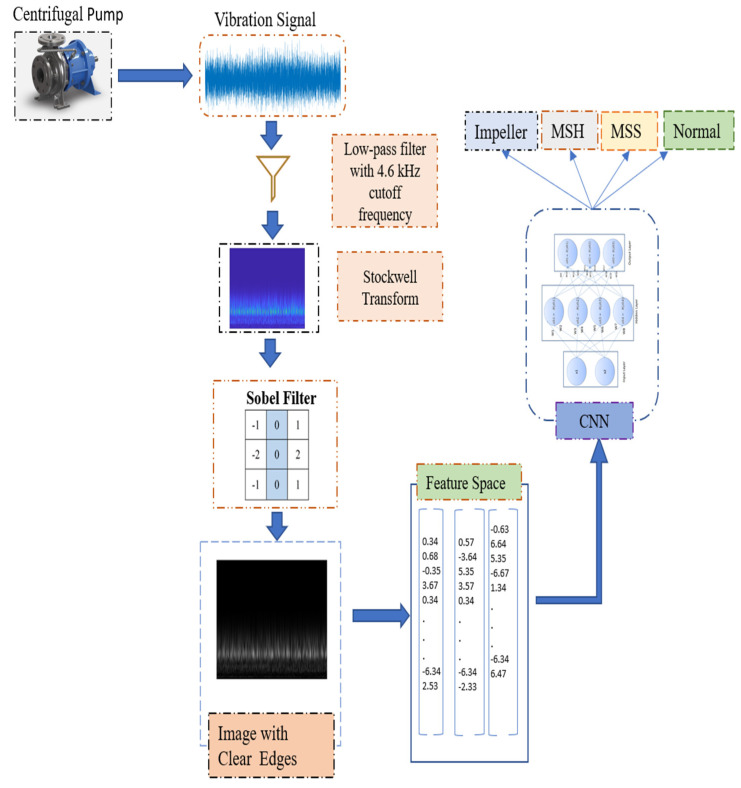
Experimental design flowchart.

The proposed method for a CP fault classification is a combination of signal processing and deep learning techniques. First, the vibration signal is taken from the sensor attached to the CP, and the signal is preprocessed by applying a low-pass filter with a 4.6 kHz cutoff frequency to obtain fault-specific frequencies [4]. After the preprocessing, we apply the S-transform, which generates a time-frequency scalogram for the time series signal. Afterward, a Sobel filter is applied to create new SobelEdge scalograms. We fed these new images (scalograms) into a CNN model that extracts latent features from the images and finally classifies these images into four classes (i.e., normal, impeller fault, mechanical seal hole, and mechanical seal scratch) using several densely connected layers. An abstract diagram of the proposed approach is depicted in Figure 1.

The proposed approach comprises the following six steps:

(1) Acquire vibration signals under different CP conditions using a data acquisition system.
(2) Extract fault-specific frequencies using a low-pass filter with a 4.6 kHz cutoff frequency [4].
(3) Generate traditional scalograms using the S-transform.
(4) Use the Sobel filter for edge extraction to generate SobelEdge Scalograms.
(5) Train a CNN classifier with SobelEdge Scalograms for the classifications Impeller Defect, Mechanical Seal Hole, Mechanical Seal Scratch, and Normal.
(6) Classify the CP vibration signal using a trained CNN classifier according to the aforementioned four classifications.

## 3. Experimental Setup and Test Rig Setup

**Figure 2:**
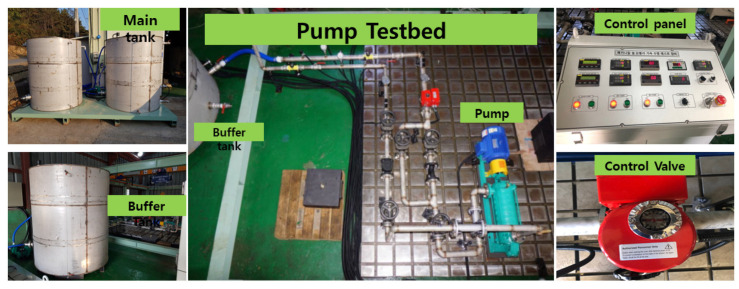
Experimental setup for collecting vibration data.

**Figure 3:**
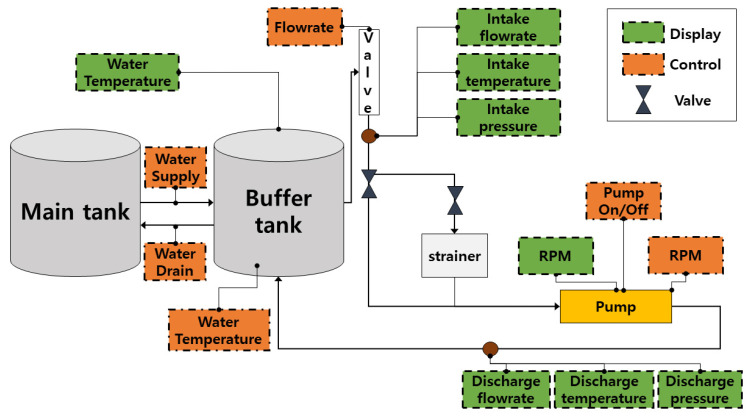
Schematic diagram of the experimental test bed.

The experiment used a CP (PMT-4008, a commonly used pump in the industry) powered by a 5.5 kW motor, as well as a control panel with an ON/OFF switch, speed controller, flow rate controller, temperature controller, water supply controller, display screens, pressure gauges, clear steel pipes, and two tanks (main tank and buffer tank). The water tank was positioned at a suitable height to maintain the net positive suction head (NPSH) at the pump inlet for the regular functioning of the CP. Figure 2 and Figure 3 illustrate the test rig configuration as well as a schematic of the system. The test rig was run to circulate water in a closed loop once the primary setup was made. Four accelerometers were used to record the CP vibration data, two of which were adhered to the pump casing, and the other two of which were installed adjacent to the mechanical seal and the impeller. Each sensor uses a separate channel to record the pump’s vibration.

The signal was then sent to a signal monitoring unit, where it was digitized by a National Instruments 9234 device. Table 1 lists the specifications of the data collection devices.

Data is collected for Impeller, MSH, MSS, and Normal for 304, 311, 315, and 317 s, respectively, with a sampling rate of 25.6 kHz. The high sample rate was maintained due to the mechanical seal excitation frequencies, which occur between the second and third modes of vibration. A total of 1247 samples, each having a length of 25,600, were obtained from the CP under various operational situations. In the current study, the pump was operated under both normal and simulated fault situations. The simulated faults include:

I. Mechanical seal faults
  - Mechanical seal hole
  - Mechanical seal scratch
II. Impeller faults.

The signals were acquired by simulating these faults, one by one. The measurement noises for the collected signals under each circumstance were estimated relative to a healthy baseline vibration signal. The mechanical seal hole, mechanical seal scratch, and impeller fault vibration signal measuring noises were determined to be −69.10, −62.07, and −63.78 dB, respectively.

### 3.1. Mechanical Seal Faults

Excessive pressure is the main cause of seal failure. The rotating portion of the mechanical seal is kept in contact with the stationary portion using a spring or combination of springs to prevent leaking from the pump during installation. These springs require a specific amount of compression pressure. When this pressure is exceeded, the mechanical seal faces are subjected to excessive pressure. This may cause overheating, and, in turn, the thin lubricating coating of liquid between the sealing faces may be converted into vapor. One of the greatest hazards to the mechanical seal is dirt. Due to the increased pressure of the springs in the absence of a lubricating coating, any dirt particles that become wedged between the sealing faces during operation can cause holes, scratches, and even harden and brittle the seal faces. These kinds of early seal failures are extremely harmful and cause the pump to fail catastrophically. In this work, hole and scratch faults were seeded in the mechanical seal and vibration signals were recorded to prevent ailments caused by these types of premature seal failures.

#### 3.1.1. Mechanical Seal Hole

**Figure 4:**
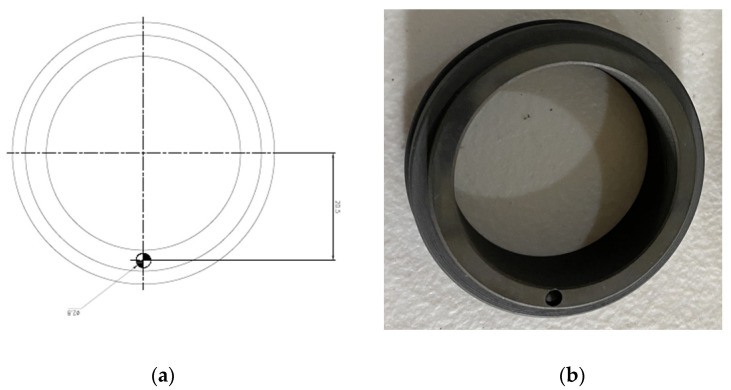
(**a**) Schematic diagram of mechanical seal hole. (**b**) Photograph of mechanical seal hole.

A mechanical seal is composed of a rotating part and a stationary part. Two seals, each with a diameter of 38 mm, were employed in this study. As seen in Figure 4, a hole was made in the rotating part of the seal, whereas the stationary part had no defects. The hole had a 2.8 mm diameter and a 2.8 mm depth. This was utilized as a faulty seal to analyze the weak fundamental fault associated with a mechanical seal hole defect.

#### 3.1.2. Mechanical Seal Scratch

**Figure 5:**
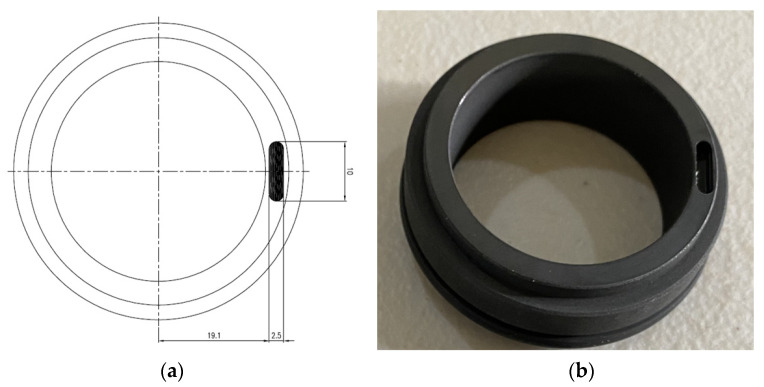
(**a**) Schematic diagram of mechanical seal scratch. (**b**) Photograph of mechanical seal scratch.

A scratch was generated in the rotating part of the mechanical seal, whereas the stationary part remained fault-free. The mechanical seal in Figure 5 has a severe fault caused by a scratch that measures 2.5 mm in diameter, 10 mm in length, and 2.8 mm in depth.

### 3.2. Impeller Fault

One common cause of an impeller malfunction is crevice corrosion. Crevice corrosion causes an uneven surface with numerous overlapping holes of various sizes that give the impression that an insect has eaten away at the impeller’s surface. Due to shear on the material, these holes may turn into significant cracks, which can cause fatigue and lead to catastrophic collapse. In this study, an impeller with a comparable issue was seeded, and vibration signals from the flawed impeller were collected.

**Figure 6:**
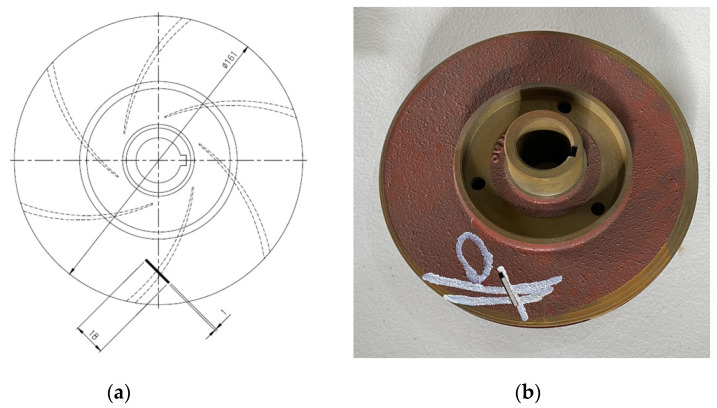
(**a**) Schematic diagram of impeller fault. (**b**) Photograph of impeller fault.

**Figure 7:**
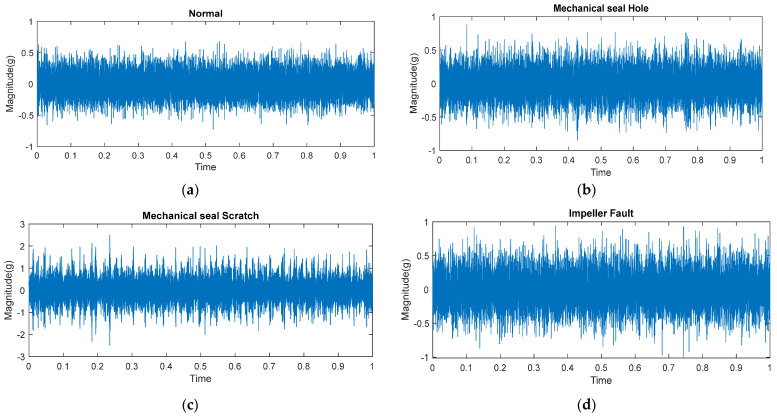
Time amplitude signals of (**a**) normal (**b**) mechanical seal hole, (**c**) mechanical seal scratch, and (**d**) impeller fault.

Three cast iron impellers 161 mm in diameter were employed in this study. The two impellers were brand new and in perfect condition. As seen in Figure 6, a defect was made in the third impeller by removing some of the metal. The defect measured 2.5 mm in diameter, 18 mm in length, and 2.8 mm in depth. Figure 7 depicts the vibration signal acquired from the damaged impeller while maintaining the functionality of all other components.

## 4. Technical Background

### 4.1. Stockwell Transform

STFT and wavelet transform components are uniquely combined in the S-transform, an encoding time-frequency spectral localization method.

The S-transform is derived as the “phase correction” of the continuous wavelet transform (CWT). CWT $W\left(\tau ,\;d\right)$ is a function of $h\left(t\right)$, which is defined by,

(1) $$W\left(\tau ,\;d\right)=\int_{-\infty }^{\infty }h\left(t\right)\omega (t-\tau ,d)dt$$

where $\omega \left(t,d\right)$ represents a scaled version of the basic mother wavelet. The resolution is governed by the dilation d, which defines the “width” of the wavelet $\omega \left(t,d\right)$. In addition to Equation (1), the mother wavelet $\omega \left(t,d\right)$ also has an admissibility requirement that it has a zero mean [13].

According to the definition, the S-transform of a function $h\left(t\right)$ is a CWT with a particular mother wavelet multiplied by the phase factor.

(2) $$S\left(\tau ,\;f\right)=e^{i2\pi f\tau }W\left(\tau ,\;d\right)$$

The mother wavelet is described as

(3) $$\omega \left(\tau ,\;f\right)=\frac{\left|f\right|}{\sqrt{2\pi }}e^{-\frac{t^{2}f^{2}}{2}}\;e^{-i2\pi f\tau }$$

Note that the frequency f  and the dilation factor d are inverses of each other.

Because the wavelet in (3) does not adhere to the strict definition of a CWT (zero mean), (2) is not a valid CWT. The explicit formula for the S-transform is,

(4) $$S\left(\tau ,\;f\right)=\int_{-\infty }^{\infty }h\left(t\right)\frac{\left|f\right|}{\sqrt{2\pi }}e^{-\frac{{\left(\tau -t\right)}^{2}f^{2}}{2}}\;e^{-i2\pi f\tau }dt$$

If the local spectrum is represented by the S-transform, the Fourier spectrum should be produced by a straightforward process of averaging the local spectra across time [12].

A signal is divided into a sequence of time-frequency slices using the S-transform, where each slice reflects the energy content of the signal at a certain frequency during a brief time period. More energy content in the signal at a specific frequency and time interval is indicated by brighter zones in a vibration signal’s S-transform.

**Figure 8:**
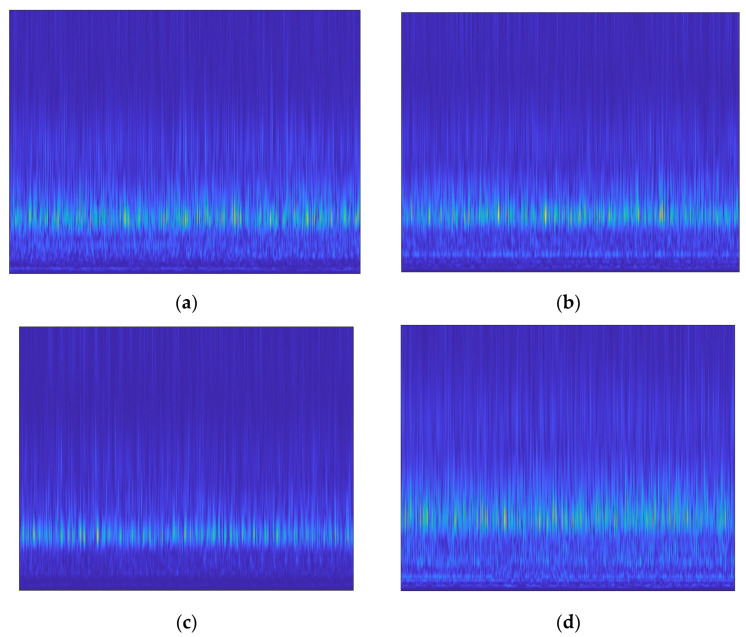
Traditional Stockwell transform scalograms of (**a**) impeller fault, (**b**) mechanical seal hole, (**c**) mechanical seal scratch, and (**d**) normal impeller and seals.

Brighter regions in the S-transform can be used to identify parts of a vibration signal that have larger amplitudes or more intense vibrations. The behavior of the system that is causing the vibration signal may be examined and understood using this information. When the CP operating conditions were changed, the scalograms computed from the CP vibrational signals with the S-transform clearly displayed various brighter areas. The S-transform scalograms of the vibration signals for each CP operating state are shown in Figure 8.

### 4.2. SobelEdge Scalograms

**Figure 9:**
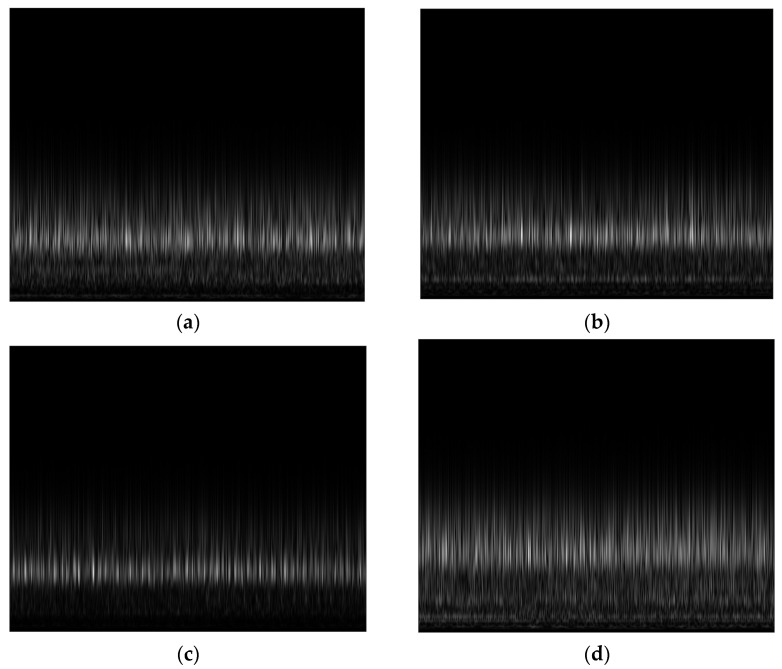
Sobel-filtered Stockwell scalogram of (**a**) impeller fault, (**b**) mechanical seal hole, (**c**) mechanical seal scratch, and (**d**) normal impeller and seals.

The vibration signal is collected from a centrifugal pump by means of sensors, and after pre-processing, the s-transform is applied. This transformation provides s-transform scalograms that contain noise, and the CNN model could not classify them accurately. To remove the noise, the Sobel filter was applied to these noisy scalograms, converting them to grayscale, making the edges clear, and reducing the amount of noise. The modified scalograms are special in that they emphasize both the time and frequency domains of the signal. Moreover, it is much simpler to discover and investigate localized phenomena such as oscillations, transient events, and frequency modulations with these scalograms. These modified scalograms are called SobelEdge scalograms. As a result, the SobelEdge scalograms are less noisy, enabling a CNN to accurately classify them. Figure 9 shows the SobelEdge scalograms that correspond to the classical scalograms seen in Figure 8.

### 4.3. Materials and Methods

After the vibration sensors are installed in the CP, we take the vibration signal from the sensor. Our model classifies the vibrational signal into four main fault states (i.e., impeller fault, MSH, MSS, and normal) to show if there is any problem in the CP. From the experiment, we noticed that this problem can be encountered by the phase difference of the vibrational signal. By applying the S-transform, phase information is added to the wavelet transform [27], providing better time-frequency resolution than STFT [28] and preserving phase information where other transformations do not [29], as well as handling non-stationary signals more effectively and exhibiting greater robustness to noise [30], making it a useful tool for signal processing applications in noisy environments.

### 4.4. Convolutional Neural Network

**Figure 10:**
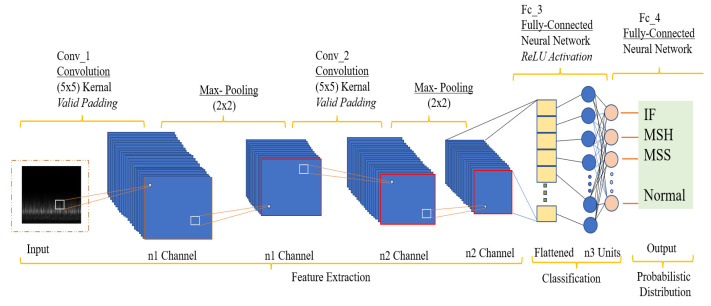
Fundamental structure of CNN.

A deep feed-forward neural network model called CNN is used to handle data having mesh-like structures [31]. CNNs are mostly used for handwritten figure identification and were initially described by Yann LeCun in 1989 [32]. Through the construction of several filters, a CNN can extract complex feature representations from input data layer by layer [33]. It integrates the sparse connections with the parameter weight-sharing technique, down-samples the data dimensions in time and space, and significantly decreases the number of training parameters to prevent the algorithm from overfitting [34]. The backpropagation (BP) technique is utilized in the CNN model to update the model’s parameters [34,35]. It is commonly used in image recognition and other comparable issues because of its high flexibility in the scaling, rotating, and translating of pictures [36,37]. Figure 10 depicts the fundamental organizational structure of a CNN [38].

CNN is a multilayer neural network [24]. One trainable feature extraction step and one classification stage make up the simplest CNN model [33]. Each stage has a particular purpose and uses both linear and nonlinear procedures. The convolutional layer, the activation layer, and the pooling layer are the three layers that make up the feature extraction step [33]. Multiple feature extraction levels can be alternately stacked to create a deep CNN network. Layer by layer, representative features are extracted from the input data using the feature extraction steps. Several densely connected layers make up the multilayer perceptron’s classification step [36]. Feature maps are sets of matrices that are used as the input and output of each layer [39]. The mathematical formula for the feedforward calculating procedure is:

(5) $$f\left(X\right)=f_{L}\left(\ldots \;f_{2}\left(f_{1}\left(X,w^{1}\right),w^{2}\right),\ldots \right),w^{L}\;$$

Here, X is the input feature, e.g., a text, image, or vibrational signal from the sensor; $w^{1},\;w^{2},\;\ldots \;w^{L}\;$ are the trainable parameters, i.e., weights and biases; $f_{1}\;,f_{2},\;\ldots \;f_{L}$ are linear or nonlinear activation functions that apply to associated layers; and $f\left(X\right)$ is the model that applies multiple activations, convolution, and pooling operations to a set of input features X, and gets the predicted label.

The CNN model consists of several layers, each serving a specific purpose in processing the input data. The initial “rescaling” layer standardizes the input images. Subsequently, three convolutional layers, “conv2d”, “conv2d_1”, and “conv2d_2”, apply convolution operations to extract relevant features, utilizing the ReLU activation function. Max pooling layers, “max_pooling2d” and “max_pooling2d_1”, reduce spatial dimensions through down sampling. The final “max_pooling2d_2” layer produces a feature map of dimensions (32, 32, 64). The “flatten” layer reshapes the feature map into a 1D vector. Two dense layers, “dense” and “dense_1”, with ReLU and Softmax activations, respectively, are responsible for classification. The model contains a total of 8,413,064 trainable parameters. The ReLU activation introduces non-linearity, while Softmax produces probability distributions for accurate classification. Through its architecture and learned parameters, the CNN model effectively processes input data and makes predictions with high accuracy. The whole work is summarized in the following Table 2.

### 4.5. Sobel Filter

The Sobel filter is a filter used in image processing to find edges. It works by combining an image with a tiny matrix called the Sobel operator. This operator calculates the gradient at each pixel to enhance the edges in the image. A Sobel filter for Stockwell scalograms is a digital filter applied to the time-frequency representation of a two-dimensional signal known as a Stockwell scalogram. By applying a complicated Fourier transform to a sliding window of the signal and then computing the magnitude of the resultant spectrum, the S-transform is used to generate the scalogram. By comparing neighboring frequency components in the scalogram, the Sobel filter is used to identify abrupt changes in the signal’s frequency content. These variations may be suggestive of significant events in the signal, such as transients or anomalies, and may be of importance for many applications, such as leak detection and condition monitoring [40].

Depending on the application and the intended output, the precise implementation of a Sobel filter for a Stockwell scalogram might vary. By comparing neighboring frequency components in the scalogram, the filter is generally intended to identify rapid changes in the signal’s frequency content. The filtered scalogram that is produced may then be further studied to find particular areas or characteristics of interest, which may be symptomatic of leaks or other problems in a fluid-carrying system [41]. The efficiency of the Sobel filter relies on the quality of the S-transform, the design of the filter, and the user’s skill in reading the resultant scalogram after filtering. Overall, Sobel filters for Stockwell scalograms are a valuable tool for detecting and evaluating changes in the frequency content of signals, and they may be used in a wide array of signal processing and condition monitoring applications [42].

## 5. Results and Discussions

It is important to build up appropriate training and testing subsets of the data in order to assess the effectiveness of our approach to fault identification. A total of 1247 Stockwell images were used in this study: 304 impeller fault images, 311 mechanical seal hole images, 315 mechanical seal scratch images, and 317 normal images. The Stockwell images were split into training and testing sets where the test size was 0.2. The testing set had 250 data samples, whereas the training set included 997 Stockwell images.

### Performance and Comparison

In this study, Stockwell images of vibration signals were calculated, and after receiving Stockwell images, a Sobel filter was applied in order to detect edges. Those grayscale images were fed into a CNN classifier. The CP’s health status was then determined by the classifier. The findings from the suggested model were more accurate and robust than those of existing state-of-the-art models. We employed several measures, such as accuracy, precision, recall, and F1 score to compare the performance of our technique with the performance of the reference methods. The following list includes the formulae used to calculate these measures.

(6) $$Accuracy\;\frac{\left(TN+TP\right)}{\left(TN+TP+FN+FP\right)}\times 100\%$$

(7) $$Precision=\frac{TP}{\left(TP+FP\right)}\times 100\%$$

(8) $$Recall=\frac{TP}{\left(TP+FN\right)}\times 100\%$$

(9) $$F_{1}-Score=\frac{2TP}{2TP+FP+FN}=2\times \frac{Precision\times Recall}{Precission+Recall}$$

where *TP* is true positives (positive samples correctly retrieved by the classifier), *TN* is true negatives (negative samples correctly retrieved by the classifier), *FP* is false positives (positive samples incorrectly retrieved by the classifier) and *FN* is false negatives (negative samples incorrectly retrieved by the classifier).

In the research conducted, K-fold cross-validation was the preferred technique for training and validating deep learning models. It also helps to reduce bias in deep learning models by providing a more accurate estimate of a model’s performance on unseen data. In the proposed study, 80% of the data were used for training while the remaining 20% of the data were used for testing the model performance. This division theory is explained by Géron A [43] for the similar size of the dataset. Utilizing five-fold validation with a confusion matrix enabled a comprehensive analysis of each model’s performance and the identification of areas for improvement. A comparison of performance metrics across different models demonstrated the superiority of this approach and validated its effectiveness in addressing the research problem.

After applying the proposed method to real-world industrial vibrational data, the suggested technique resulted in accuracy, precision, recall, and F1 scores of 99.68%, 99.65%, 99.68%, and 99.66%, respectively. Table 3 presents the results obtained from the proposed method and the reference methods. It can be seen from Table 4 that the proposed method outperformed the reference methods in terms of classification accuracy. The overperformance of the proposed method can be explained as follows. The proposed method outperforms reference models across all performance parameters because of its core idea of using SobelEdge scalograms for vibrational signals, which is useful in improved time-frequency resolution, ability to capture transients, reduced interference, and ease of interpretation.

To evaluate the effectiveness of the proposed model, the proposed model was compared with two other relevant models used for similar purposes. The first model is a fault diagnosis method by Weifang Sun et al. [43] that uses converted 2D vibrational signal matrices, a mean curvature algorithm to eliminate interference, a histogram of oriented gradients (HOG) features for fault feature extraction and a support vector machine for automatic fault classification. After applying the steps presented by Weifang Sun et al. [44] to our dataset, the method resulted in accuracy, precision, recall, and F1 scores of 91.60%, 91.56%, 91.60%, and 91.43%, respectively. Underperformance was expected because the vibrational signals are heavily affected by noise. Furthermore, these scalograms do not carry energy distribution as well because they lack phase information in the vibrational signals.

The method proposed by Gabor et al. [45] uses an improved STFT algorithm and image processing techniques, including differential and moving average predictive tracking algorithms, with the potential for real-time condition monitoring based on a basic vibration measurement. The method was tested and validated with simulated signals and transient measurements on rotating machines, demonstrating efficient and accurate analysis. After applying the steps proposed in [45] to our dataset, the method resulted in accuracy, precision, recall, and F1 scores of 89.61%, 91.26%, 89.61%, and 88.96%, respectively. The underperformance outcomes were due to the use of STFT scalograms for vibrational signals, which carry certain limitations including poor resolution, interference, computational complexity, sensitivity to window selection, and inability to catch transients.

**Figure 11:**
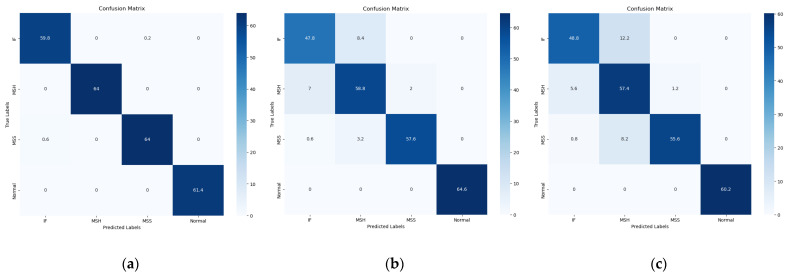
Confusion matrices of (**a**) proposed model, (**b**) Weifang Sun et al. [44] (**c**) Gabor et al. [45].

**Figure 12:**
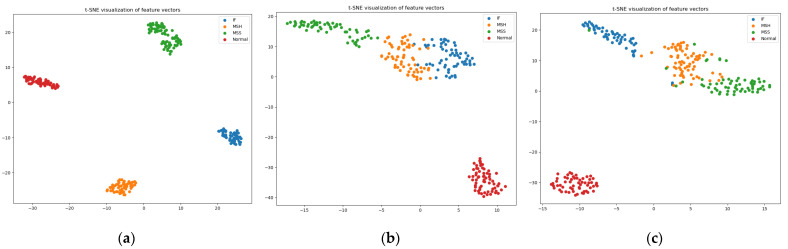
t-SNE of (**a**) proposed model, (**b**) Weifang Sun et al. [44], and (**c**) Gabor et al. [45].

Table 3 compares the proposed method with the Weifang et al., and Gabor et al., models in terms of all performance parameters for each fault class. Additionally, Table 4 compares the average performance parameters of the three models. Moreover, the comparison of confusion matrices in Figure 11, provides valuable insights into the classification performance of each model. The observed results indicate that among all the models considered, only the proposed model achieved precise classification for the normal class. In contrast, the remaining three classes were accurately classified only by the proposed model. This discrepancy can be attributed to the fact that the vibrational signals used in the referenced models are susceptible to significant noise interference, thereby compromising their classification accuracy. Additionally, the reference scalograms employed in these models exhibit limitations in terms of capturing the energy distribution due to the absence of phase information in the underlying vibrational signals. To further illustrate the discriminative capabilities of the proposed study, t-SNE plots presented in Figure 12 are analyzed. The t-SNE plots demonstrate that, similar to the reference models (Weifang et al., and Gabor et al., models), the proposed study effectively discriminates the faulty signals. However, the proposed model outperforms the referenced models by significantly enhancing the discrimination of each distinct type of fault. This remarkable improvement is a noteworthy contribution of the proposed model, enabling more precise and accurate fault classification.

## 6. Conclusions

This research presented a novel approach for fault classification in centrifugal pumps using signal processing and deep learning techniques. The proposed approach employed a vibration sensor that recorded raw frequencies, which were passed from a low-pass filter to attain fault-specific frequencies and are further processed using the Stockwell Transform and Sobel filtering to generate new scalograms called “SobelEdge scalograms” for classification using a CNN model. The proposed approach has the potential to provide an accurate and efficient solution for fault diagnosis, which could help to prevent pump failures and reduce maintenance costs in industrial applications. The findings of this study could be useful for researchers and practitioners working in the field of fault diagnosis and classification in centrifugal pumps. The overall accuracy of the proposed model is 99.68%. Furthermore, the proposed method was able to classify all four operation states, unlike state-of-the-art methods that have failed to classify the IF, MSH, and MSS precisely. Our aim for upcoming research is to improve our method so that it can classify all possible faults of CPs. Additionally, we intend to improve our method such that it can monitor the health condition of all parts of CPs.

## Figures and Tables

**Table 1:** Specifications of data acquisition devices.

Device Name	Specification
Accelerometer (622b01)	Range of frequency: 0.4 → 10 kHz Sensitivity: 100 mV/g (10.2 mV/g (ms^−2^)) ± 5%
DAQ System (NI9234)	Range of frequency: 0 → 13.1 MHz Generator: Four analog input channels 24-bit ADC resolution

**Table 2:** The CNN architecture.

Layer (Type)	Output Shape	Param No.	Activation Function
rescaling (Rescaling)	(None, 256, 256, 1)	0	-
conv2d (Conv2D)	(None, 256, 256, 16)	160	ReLU/-
max_pooling2d (Maxpooling2D)	(None, 128, 128, 16)	0	-
conv2d_1 (Conv2D)	(None, 128, 128, 32)	4640	ReLU/-
max_pooling2d_1 (Maxpooling2D)	(None, 64, 64, 32)	0	-
conv2d_2 (Conv2D)	(None, 64, 64, 64)	18496	ReLU/-
max_pooling2d_2 (Maxpooling2D)	(None, 32, 32, 64)	0	-
flatten (Flatten)	(None, 65536)	0	-
dense (Dense)	(None, 128)	8388736	ReLU/-
dense (Dense)	(None, 4)	516	Softmax

**Table 3:** Comparison of results of the proposed model with those of Weifang Sun et al. [44] and Gabor et al. [45].

	Accuracy				Precision				F1 Score				Recall			
Model	IF	MSH	MSS	Nomal	IF	MSH	MSS	Nomal	IF	MSH	MSS	Nomal	IF	MSH	MSS	Nomal
Proposed	99.62	100	99.10	100	98.93	100	99.69	100	99.27	100	99.39	100	99.62	100	99.10	100
Weifang Sun	85.58	87.02	93.79	100	86.84	83.59	96.60	100	85.58	87.02	93.79	100	85.60	84.95	95.15	100
Gabor	83.01	89.58	85.84	100	90.54	76.53	97.98	100	83.02	89.58	85.84	100	83.10	81.47	91.32	100

**Table 4:** Comparison of average results of the proposed model with those of Weifang Sun et al. [44] and Gabor et al. [45].

Model	Accuracy	Precision	Recall	F1 Score
Proposed	99.68	98.93	100	99.69
Weifang Sun	91.60	91.57	91.60	91.43
Gabor	89.61	91.26	89.61	88.96

## Data Availability

The data is from the industry. Due to the privacy policy of the industry the data is not available publicly.

## Nomenclature

AI	Artificial intelligence
CBM	Condition-based monitoring
CPs	Centrifugal pumps
CNN	Convolutional neural network
FD	Fault diagnosis
FT	Fourier transform STFT—Short-term Fourier transform
S	transform: Stockwell transform
TFD	Time-frequency-domain
WT	Wavelet transform
ω	Wavelet
$w^{L}$	Set of trainable parameters at L^th^ layer
$f_{L}$	Activation function at Lth layer

## References

[1] Vogelesang H. (2008). An introduction to energy consumption in pumps. World Pumps..

[2] Shankar V.K.A., Umashankar S., Paramasivam S., Hanigovszki N. (2016). A comprehensive review on energy efficiency enhancement initiatives in centrifugal pumping system. Appl. Energy.

[3] Sunal C.E., Dyo V., Velisavljevic V. (2022). Review of Machine Learning Based Fault Detection for Centrifugal Pump Induction Motors. IEEE Access.

[4] Ahmad Z., Nguyen T.-K., Ahmad S., Nguyen C.D., Kim J.-M. (2021). Multistage Centrifugal Pump Fault Diagnosis Using Informative Ratio Principal Component Analysis. Sensors.

[5] Lei Y. (2016). Intelligent Fault Diagnosis and Remaining Useful Life Prediction of Rotating Machinery.

[6] Rapur J.S., Tiwari R. (2019). Experimental fault diagnosis for known and unseen operating conditions of centrifugal pumps using MSVM and WPT based analyses. Measurement.

[7] Panda A.K., Rapur J.S., Tiwari R. (2018). Prediction of flow blockages and impending cavitation in centrifugal pumps using Support Vector Machine (SVM) algorithms based on vibration measurements. Measurement.

[8] Shafiullah A.M.A. (2018). S-transform based FFNN approach for distribution grids fault detection and classification. IEEE Access.

[9] Zhang X., Hu Y., Deng J., Xu H., Wen H. (2022). Feature Engineering and Artificial Intelligence-Supported Approaches Used for Electric Powertrain Fault Diagnosis: A Review. IEEE Access.

[10] Satpathi K., Yeap Y.M., Ukil A., Geddada N. (2018). Short-Time Fourier Transform Based Transient Analysis of VSC Interfaced Point-to-Point DC System. IEEE Trans. Ind. Electron..

[11] Khan U.N. Signal Processing Techniques Used In. http://eeeic.org/proc/papers/23.pdf.

[12] Stockwell R.G., Mansinha L., Lowe R.P. (1996). Localization of the complex spectrum: The S transform. IEEE Trans. Signal Process..

[13] Daubechies I. (1990). The Wavelet Transform, Time-Frequency Localization and Signal Analysis. IEEE Trans. Inf. Theory.

[14] Peng Z.K., Chu F.L. (2004). Application of the wavelet transform in machine condition monitoring and fault diagnostics: A review with bibliography. Mech. Syst. Signal Process..

[15] Wang C., Gao R.X. Wavelet transform with spectral post-processing for enhanced feature extraction. Proceedings of the 19th IEEE Instrumentation and Measurement Technology Conference (IEEE Cat. No.00CH37276).

[16] Zhang J.-Y., Cui L.-L., Yao G.-Y., Gao L.-X. Research on the selection of wavelet function for the feature extraction of shock fault in the bearing diagnosis. Proceedings of the 2007 International Conference on Wavelet Analysis and Pattern Recognition.

[17] Wan S.-T., Lv L.-Y. The fault diagnosis method of rolling bearing based on wavelet packet transform and zooming envelope analysis. Proceedings of the 2007 International Conference on Wavelet Analysis and Pattern Recognition.

[18] Lee B.Y., Tarng Y.S. (1999). Application of the Discrete Wavelet Transform to the Monitoring of Tool Failure in End Milling Using the Spindle Motor Current. Int. J. Adv. Manuf. Technol..

[19] Chang J., Kim M., Min K. (2002). Detection of misfire and knock in spark ignition engines by wavelet transform of engine block vibration signals. Meas. Sci. Technol..

[20] Goumas S.K., Zervakis M.E., Stavrakakis G.S. (2002). Classification of washing machines vibration signals using discrete wavelet analysis for feature extraction. IEEE Trans. Instrum. Meas..

[21] Cristaldi L., Lazzaroni M., Monti A., Ponci F. (2004). A neurofuzzy application for AC motor drives monitoring system. IEEE Trans. Instrum. Meas..

[22] Yan R., Gao R.X. (2009). Energy-based feature extraction for defect diagnosis in rotary machines. IEEE Trans. Instrum. Meas..

[23] Delgado M., Cirrincione G., Garc A., Ortega J.A., Henao H. Accurate bearing faults classification based on statistical-time features, curvilinear component analysis and neural networks. Proceedings of the IECON 2012—38th Annual Conference on IEEE Industrial Electronics Society.

[24] Xia M., Li T., Xu L., Liu L., de Silva C.W. (2017). Fault Diagnosis for Rotating Machinery Using Multiple Sensors and Convolutional Neural Networks. IEEE/ASME Trans. Mechatron..

[25] Ahmad Z., Rai A., Hasan M.J., Kim C.H., Kim J.-M. (2021). A Novel Framework for Centrifugal Pump Fault Diagnosis by Selecting Fault Characteristic Coefficients of Walsh Transform and Cosine Linear Discriminant Analysis. IEEE Access.

[26] Ahmad S., Ahmad Z., Kim J.-M. (2022). A Centrifugal Pump Fault Diagnosis Framework Based on Supervised Contrastive Learning. Sensors.

[27] Djurovi’cdjurovi I., Sejdi E., Jiang J. (2008). Frequency-based window width optimization for S-transform. Int. J. Electron. Commun. (AEÜ).

[28] Su D., Li K., Shi N. (2021). Power quality disturbances recognition using modified s-transform based on optimally concentrated window with integration of renewable energy. Sustainability.

[29] Boashash B. (2015). Time-Frequency Signal Analysis and Processing: A Comprehensive Reference.

[30] Bajaj A., Kumar S. (2020). A robust approach to denoise ECG signals based on fractional Stockwell transform. Biomed. Signal Process. Control.

[31] Goodfellow I., Bengio Y., Courville A. (2016). Deep Learning.

[32] LeCun Y., Bottou L., Bengio Y., Haffner P. (1998). Gradient-based learning applied to document recognition. Proc. IEEE.

[33] Zhang W., Li C., Peng G., Chen Y., Zhang Z. (2018). A deep convolutional neural network with new training methods for bearing fault diagnosis under noisy environment and different working load. Mech. Syst. Signal Process..

[34] Lecun Y., Bengio Y., Hinton G. (2015). Deep learning. Nature.

[35] Schmidhuber J. (2015). Deep learning in neural networks: An overview. Neural Netw..

[36] Zhang W., Peng G., Li C. (2017). Bearings Fault Diagnosis Based on Convolutional Neural Networks with 2-D Representation of Vibration Signals as Input. MATEC Web Conf..

[37] Verstraete D., Ferrada A., Droguett E.L., Meruane V., Modarres M. (2017). Deep learning enabled fault diagnosis using time-frequency image analysis of rolling element bearings. Shock Vib..

[38] (2018). Understanding of Convolutional Neural Network (CNN)—Deep Learning. Medium.

[39] LeCun Y., Kavukcuoglu K., Farabet C. Convolutional networks and applications in vision. Proceedings of the 2010 IEEE International Symposium on Circuits and Systems.

[40] Gong J., Yang X., Pan F., Liu W., Zhou F. (2021). An Integrated Fault Diagnosis Method for Rotating Machinery Based on Improved Multivariate Multiscale Amplitude-Aware Permutation Entropy and Uniform Phase Empirical Mode Decomposition. Shock Vib..

[41] Ravivarma G., Gavaskar K., Malathi D., Asha K.G., Ashok B., Aarthi S. (2021). Implementation of Sobel operator based image edge detection on FPGA. Mater. Today Proc..

[42] Dong X., Li J., Wu J., Liu J. (2020). A Window Detection Algorithm for Remote Laser Gas Leakage Detection System. Procedia Comput. Sci..

[43] Géron A. (2022). Hands-On Machine Learning with Scikit-LEARN, Keras, and TensorFlow.

[44] Sun W., Cao X. (2020). Curvature enhanced bearing fault diagnosis method using 2D vibration signal. J. Mech. Sci. Technol..

[45] Manhertz G., Bereczky A. (2021). STFT spectrogram based hybrid evaluation method for rotating machine transient vibration analysis. Mech. Syst. Signal Process..

